# Experimental manipulation of sexual traits in barn swallow populations—No evidence for divergent sexual selection

**DOI:** 10.1111/evo.14505

**Published:** 2022-07-26

**Authors:** Jan T. Lifjeld

**Affiliations:** ^1^ Natural History Museum University of Oslo Oslo Norway

**Keywords:** longitudinal analysis, mate choice, paternity, phenotype manipulation

## Abstract

Safran et al. (2016a) manipulated two sexual traits (ventral plumage coloration and tail streamer length) in male barn swallows (*Hirundo rustica*) and reported divergent effects on paternity change between two study populations, in Colorado and Israel. They concluded that geographical variation in the two phenotypic traits is maintained by divergent sexual selection. However, the response variable they used, the longitudinal change in paternity from a pre‐treatment clutch to a post‐treatment clutch, does not reflect an unbiased effect of the treatment. Here, I show that the magnitude of the change in paternity is influenced by variation in the initial paternity score among the treatment groups, which is presumably due to stochastic variation from low sample sizes in the treatment groups. When the bias was accounted for in re‐analyses of the Israeli dataset, the statistical significance of one of two treatment effects disappeared. Similar re‐analyses of the American population were not possible due to inaccessibility of raw data for individual clutches, but an assessment of the mean scores indicates that the two significant treatment effects in this population were similarly biased in their initial paternity scores. The conclusion of divergent sexual selection on male phenotypic traits between the two populations does not seem to be supported.

Safran et al. (2016a) reported a field experiment designed to test the role of male secondary sexual traits for paternity success in a socially monogamous passerine bird with frequent extrapair fertilization, the barn swallow *Hirundo rustica*. The study manipulated male ventral plumage coloration and the length of the outermost tail feathers (streamers) in two widely separated study populations; in Colorado, USA, and in Israel (the results from the Israeli population were also published previously by Vortman et al. 2013a). The authors found that the phenotypic manipulations caused different paternity changes in the two populations, and they interpreted the results as evidence for divergent sexual selection maintaining geographical variation in these traits.

The experimental procedure was to manipulate male phenotypes after the first clutch was laid, then remove the clutch, and let the pair initiate a second clutch. Molecular paternity analysis identified how many eggs in the two clutches were sired by the male nest owner. The response to the treatment was measured as the change in paternity from the first to the second clutch. Male phenotype was manipulated in five ways: darkened plumage, streamer elongation, streamer shortening, and the combined treatment of darkened plumage with either elongated or shortened streamers. The experiment also had a control group with no phenotype manipulations. In the American population, males with a darkened plumage and males with shortened streamers showed a significant increase in paternity, whereas the four other groups (including controls) showed no significant changes. In the Israeli population, males with the combination of darkened plumage and elongated streamers showed increased paternity, while males with the combination of darkened plumage and shortened streamers had a significant decrease in paternity. The other groups, with a single manipulation, or no manipulation (controls), showed no significant change in paternity. When analyzing the data from the two populations together, Safran et al. (2016a) found a significant interaction of country and treatment. This led to the conclusion that there is divergent sexual selection on the two male traits and that sexual selection favors different combinations of the same traits in the two populations. Vortman et al. (2013a) also concluded that sexual selection acts specifically on multiple signals in the Israeli population, favoring the combination of dark plumage and long streamers as a mechanism of reproductive isolation from nearby divergent populations of other subspecies.

I find the use of the longitudinal change in paternity as the response variable statistically questionable. The paternity change is not only an effect of the treatment, but is influenced by variation in the initial paternity score, that is, before the manipulation of male phenotypes. This non‐treatment effect is visualized in Figure 1, for both populations and all three proxies for paternity used by Safran et al. (2016a): the proportion of withinpair young (WPY) in the clutch, the number of WPY, and the number of extrapair young (EPY), that is, eggs not sired by the resident male. In all cases, the paternity score for the first clutch explained more than 60% of the variation in paternity change among the treatment groups. Hence, the measured responses cannot be attributed to post‐treatment effects alone.

**Figure 1:**
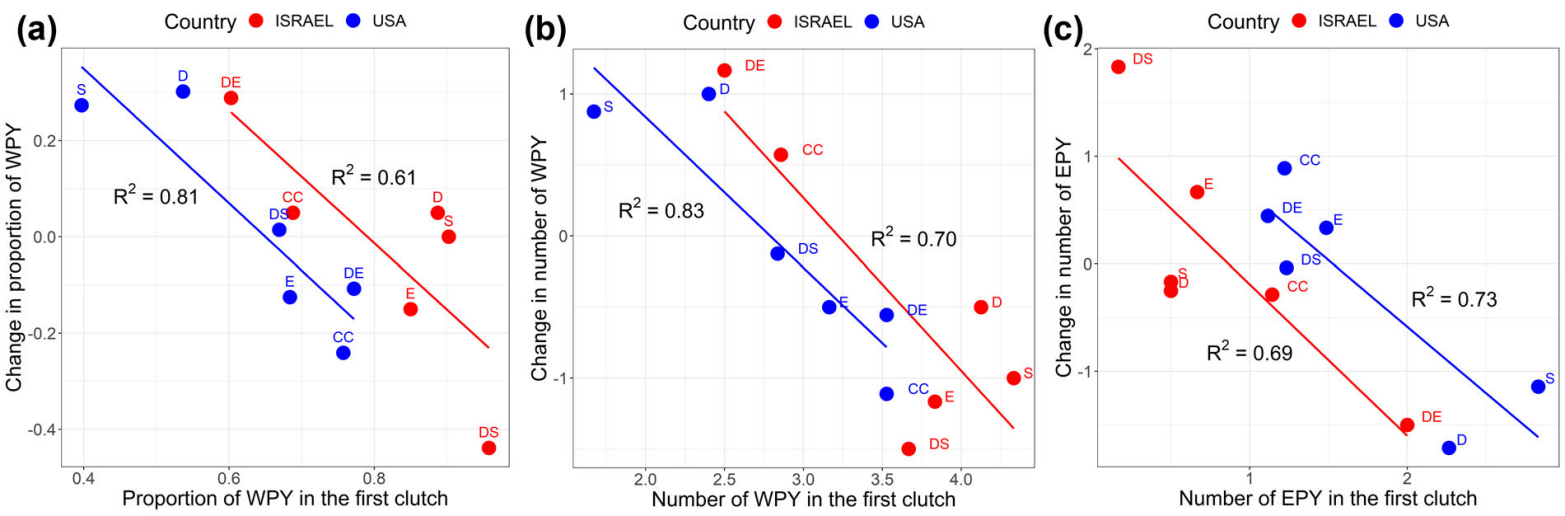
Treatment effects in the two phenotype manipulation experiments (in Colorado, USA, and Israel) on male barn swallows reported by Safran et al. (2016a). The paternity change for the six treatment groups is plotted as a function of paternity in the premanipulation clutch for each of three paternity measures: (a) proportion of withinpair young (WPY) in the clutch, (b) number of WPY, and (c) number of extrapair young (EPY). The treatment groups are annotated as D = darkened plumage, S = shortened streamers, E = elongated streamers, DS = darkened plumage and shortened streamers, DE = darkened plumage and elongated streamers, and CC = controls. Linear regression lines are indicated for each population with R^2^ for the relationships. The group means were calculated from Vortman et al. (2013b) for the Israeli population and from Safran et al. (2016b) for the paternity changes in the American population. The group means for the first clutches in the American population were fitted by eye from Figure 1 in Safran et al. (2016a).

The variation in paternity of first clutches among experimental groups does not necessarily imply any bias in the initial allocation of males to the different treatments. Instead, the variation is probably due to the small sample sizes with large standard errors around the mean estimates, as exemplified by the paternity data for the Israeli population (Fig. 2, raw data from Vortman et al. 2013b). Treatment groups contained only six to nine males in the American population and six to eight males in the Israeli population. A randomization test of the paternity data from the Israeli population, sampling six clutches at random from first and second clutches, revealed negative correlation coefficients in the range of −0.65 to −0.70 for the relationship between paternity change and initial paternity (Supporting Information). It suggests that the observed negative relationships in Figure 1 just follow from a stochastic variation in mean estimates when the group sample size is small.

**Figure 2:**
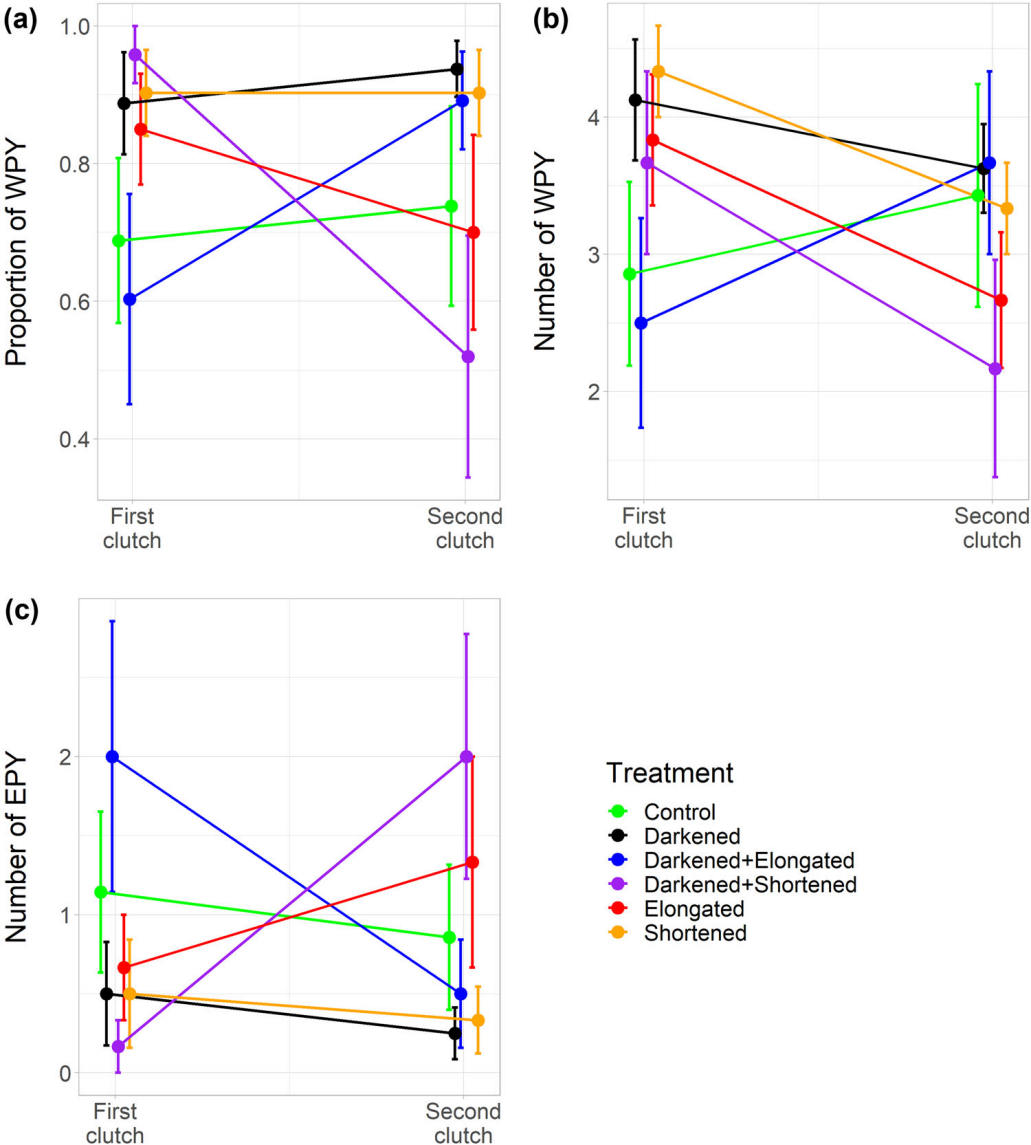
Paternity scores (mean ± SE) in first and second clutches of the six experimental groups of male barn swallows in the Israeli population reported by Vortman et al. (2013a, 2013b). The three panels show the three different proxies for paternity analyzed by Safran et al. (2016a): (a) the proportion of within‐pair young (WPY), (b) the number of WPY, and (c) the number of extrapair young (EPY).

The error bars for the group means in Figure 2 are large and overlapping, both for first and second clutches, which indicates no apparent cross‐sectional group differences either before or after treatment. Figure 2 also reveals how the two groups with a reported significant change in paternity in the Israeli population were biased by differences in initial paternity. The “Darkened + Shortened” group had a significant decrease in paternity, but it also had almost full paternity in the first clutch with only one egg lost to extrapair paternity across six nests. The potential for an increase in paternity is, therefore, extremely marginal and a decrease is more likely. Similarly, the “Darkened + Elongated” group had a significant increase in paternity, but it started out with the lowest paternity score for the first clutches of all groups, and thus, an increase is expected. Other groups ended up with a higher mean paternity score after treatment (i.e., in second clutches), which does not indicate any strong treatment effect for the “Darkened + Elongated” group.

A statistical test must, therefore, be used to account for the variation in the initial paternity score among the test groups. I re‐analyzed the data from the Israeli population in two different ways. First, I performed a randomization test comparing the observed change in paternity for a given treatment group against a random distribution of possible paternity changes for that group, using paternity data for all males in the data set (N = 39). More specifically, I sampled *n* of the 39 second clutches, where *n* is the sample size of the given treatment group, calculated the mean paternity score for the sampled clutches, and subtracted the mean observed paternity for the first clutches of the treatment group. This gives a randomly expected paternity change. The procedure was repeated 9999 times, which resulted in a collection of 10,000 randomly generated paternity changes for the particular treatment group. The rank of the observed paternity change within the ranked distribution of random changes gives the one‐tailed probability of the observed change in the direction predicted by the type of treatment. Table 1 summarizes the results of the randomization tests for the three paternity proxies. Only the “Darkened + Shortened” group had a significant change (decrease) in paternity scores. This means that the decrease was significantly larger than expected from the distribution of random changes. While Safran et al. (2016a) and Vortman et al. (2013a) concluded that the “Darkened + Elongated” group also had a significant paternity change (increase), the randomization tests revealed no statistical significance for any of the three paternity proxies for that group, nor for any of the other groups (Table 1).

**Table 1:** Treatment effects in the Israeli population based on randomization tests of expected paternity changes for each treatment group (for details see text)

		Randomization tests (one‐tailed *p*)		
Treatment group (sample size)	Predicted paternity change	Prop. WPY	No. WPY	No. EPY
Darkened (N = 8)	Increase	0.061	0.22	0.083
Elongated (N = 6)	Increase	0.79	0.84	0.88
Shortened (N = 6)	Decrease	0.84	0.65	0.89
Darkened & Elongated (N = 6)	Increase	0.21	0.25	0.31
Darkened & Shortened (N = 6)	Increase^a^	0.99	0.97	0.99
	Decrease^a^	**0.016**	0.055	**0.018**
Control (N = 7)	Increase^a^	0.71	0.38	0.61
	Decrease^a^	0.31	0.72	0.52

^a^ Predicted change can go either way.

A predicted increase in paternity implies an increase in the proportion and number of withinpair young (WPY) and a decrease in the number of extrapair young (EPY). Significant treatment effects are indicated in bold.

In the second statistical approach, I ran a generalized linear mixed model of the variation in clutch paternity expressed as the number of WPY over clutch size (i.e., WPY + EPY) with a binomial error distribution. I treated clutch identity and male identity as random factors, and analyzed the paternity of the first brood and the interaction term between the two broods (i.e., the slope) as fixed factors for each treatment group. The control group was used as the reference group, with paternity of the first clutch as the reference level. The results were similar to the randomization test: there was a significant paternity decrease for the “Darkened + Shortened” group, but no other significant changes for any of the other treatments (Table 2). Taken together, these two tests show that the “Darkened + Shortened” males had a significant paternity decrease, both when compared to the control males (Table 2) and when compared to the overall changes for all males in the dataset (Table 1). However, the paternity increase for the “Darkened + Elongated” males was not confirmed.

**Table 2:** Generalized linear mixed model on the variation in paternity among treatment groups in the Israeli population

Fixed effect	Estimate	SE	z	P
Intercept	1.3356	0.8151	1.639	0.101
Paternity change (reference level = first clutch)	0.4507	0.8937	0.504	0.614
Paternity first clutch				
Darkened	1.6074	1.2000	1.339	0.180
Elongated	1.0588	1.2289	0.862	0.389
Shortened	1.7291	1.3030	1.327	0.185
Darkened & Elongated	−0.7863	1.1541	‐0.681	0.496
Darkened & Shortened	2.6994	1.5736	1.756	0.079
Paternity change (interaction)				
Darkened	0.1904	1.4390	0.132	0.895
Elongated	−1.6508	1.3180	−1.253	0.210
Shortened	−0.3651	1.5133	−0.241	0.809
Darkened & Elongated	1.9013	1.3735	1.384	0.166
Darkened & Shortened	−4.3269	1.6248	−2.663	**0.0077**

Dependent variable is the paternity proportion expressed as the number of eggs sired (WPY) over the total number of eggs in the clutch (WPY + EPY) and a binomial error distribution. The reference group is the control group. Random factors: male identity; clutch identity. Significant fixed effects are indicated in bold.

Unfortunately, a similar re‐examination of the American population is not feasible due to lack of access to the correct raw data to date. The initial data file uploaded to the Dryad Data Repository (Safran et al. 2016b) only gives the calculated paternity change for each male, not the number of eggs sired in each clutch. A revised raw data file uploaded on May 18, 2021 contains several inconsistencies and cannot resolve this issue. However, an assessment of the paternity patterns for the American population depicted in Figure 1 can give some indication as to whether the reported significant paternity increases for the “Darkened” and the “Shortened” groups make statistical sense. Clearly, the “Darkened” and “Shortened” groups had the largest increases in paternity (see the high scores on the y‐axis in Fig. 1 A and B) and corresponding decreases in the number of EPY (low scores on the *y*‐axis in Fig. 1C). But they also had the lowest paternity and the highest number of EPY in the first clutch, and therefore, a larger paternity increase would be expected than for the other groups. It is also evident that the control group had relatively high paternity scores for the first clutch and the largest decrease in paternity among all groups. A comparison of the changes, especially with the control group, would, therefore, yield a false impression of different treatment effects, since the initial paternity scores were so different. Safran et al. (2016a) made the point that there was no statistically significant variation in paternity among groups for the first clutches, but they did not present a similar test for the second clutches. Any treatment that is predicted to increase or decrease paternity should lead to larger among‐group heterogeneity in paternity scores for the second clutches. Overall, the initial paternity scores explained more of the variation in paternity changes among the treatment groups in the American population than they did in the Israeli population (cf. the *R* ^2^‐values of the correlations in Fig. 1). This suggests that a statistical re‐analysis of the American population with correction for the variation in initial paternity would remove more of the among‐group variance in paternity change, and thus make any significant treatment effects less likely.

I conclude that the statistical approach used in the study by Safran et al. (2016a) casts doubts about their conclusions. In the Israeli population, only the “Darkened + Shortened” group showed a significant decrease in paternity, whereas there was no evidence for a paternity increase for the “Darkened + Elongated” group. The paternity increases for “Darkened” and “Shortened” males in the American population remain elusive, leaving the issue of divergent sexual selection between the two populations inconclusive.

In general, the use of a longitudinal response variable in experiments may seem appealing because it measures responses within the individual and is not concerned with variation among individuals. However, researchers should always be aware of potential biases due to various sources of error. Large measurement errors or low repeatability could lead to biases like the well‐known “regression to the mean” effect, where more extreme scores tend to move toward the sample mean when repeated (Galton 1886; Kelly and Price 2005). In the current case, low sample sizes in each treatment group gave large errors for the estimated group means, and the groups with the more extreme initial scores tended to show the largest changes as a statistical artifact. In a longitudinal experiment, unbiased groups can be ensured by randomly allocating subjects with similar initial scores to different treatment groups. Such a randomized‐block design was successfully adopted in the famous tail‐manipulation experiment on long‐tailed widowbirds *Euplectes progne* (Andersson 1982), where allocation of males to different treatments was done within groups of males with similar pre‐treatment mating success. Alternatively, if information about the initial score is unknown at the time of treatment (e.g., when subsequent paternity testing is required), researchers need to check for any pre‐treatment bias among experimental groups post‐hoc and correct for them accordingly in the statistical analysis as suggested here.

## AUTHOR CONTRIBUTIONS

JTL analyzed the data and wrote the article.

Associate Editor: R. Fuller

Handling Editor: T. Chapman

## Supporting information

Supplementary Fig. S1.docx. Random distribution of the relationship between paternity change and the paternity score for first clutch in the Israeli population for a sample size of 6 males.
Supplementary Table S1.xlsx: The random distributions from which the P‐values in Table 1 were derived.
R Script for GLMM in Table 2.docx: The R code for the analysis in Table 2.
Israel_data.csv: The data for the R script derived from Vortman et al. (2013b).

## Data Availability

Data are available on Dryad with the original studies (https://doi.org/10.5061/dryad.5865f and https://doi.org/10.5061/dryad.g8n63).
