# Supplementary material for: Experimental manipulation of sexual traits in barn swallow populations—No evidence for divergent sexual selection
Source: Evolution. 2022 Jul 26;76(9):2199–203. doi: 10.1111/evo.14505 (PMC9545097; doi:10.1111/evo.14505)

**Supporting Information**

Supplementary Figure S1. Random distribution of the relationship between paternity change and the paternity score for first clutch in the Israeli population for a sample group of six males. The randomizations were performed by sampling 6 of the 39 first clutches and 6 of the 39 second clutches at random, calculating the mean paternity for each sample and the difference between them. The procedure was performed 10 000 times for each of the three paternity measures. The three panels give the distribution of the 10 000 randomized data points for the proportion of WPY (left), the number of WPY (middle) and the number of EPY. The linear regression line (red) and the correlation coefficient are indicated.


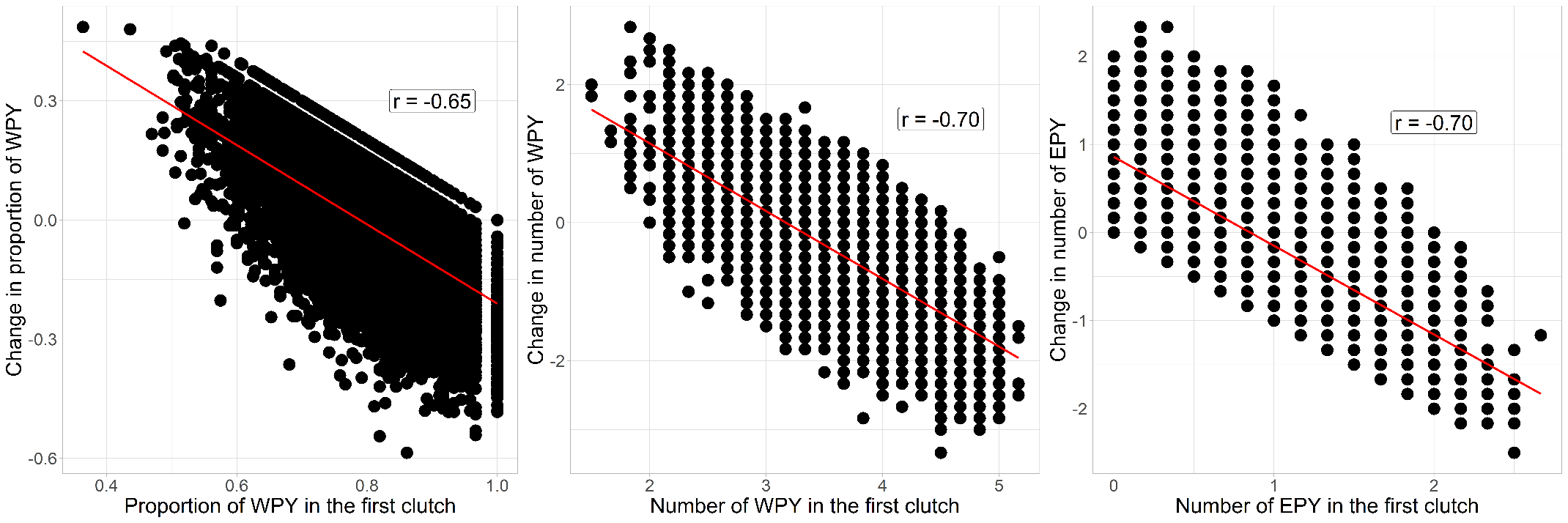

Supplement: Supplementary file 1 — Supplementary Fig. S1.docx. Random distribution of the relationship between paternity change and the paternity score for first clutch in the Israeli population for a sample size of 6 males. Supplementary Table S1.xlsx: The random distributions from which the P‐values in Table 1 were derived. R Script for GLMM in Table 2.docx: The R code for the analysis in Table 2. Israel_data.csv: The data for the R script derived from Vortman et al. (2013b). [file EVO-76-2199-s001.zip › evo14505-sup-0001-figureS1.docx]
