# Supplementary material for: Experimental manipulation of sexual traits in barn swallow populations—No evidence for divergent sexual selection
Source: Evolution. 2022 Jul 26;76(9):2199–203. doi: 10.1111/evo.14505 (PMC9545097; doi:10.1111/evo.14505)
Supplement: Supplementary file 1 — Supplementary Fig. S1.docx. Random distribution of the relationship between paternity change and the paternity score for first clutch in the Israeli population for a sample size of 6 males. Supplementary Table S1.xlsx: The random distributions from which the P‐values in Table 1 were derived. R Script for GLMM in Table 2.docx: The R code for the analysis in Table 2. Israel_data.csv: The data for the R script derived from Vortman et al. (2013b). [file EVO-76-2199-s001.zip › evo14505-sup-0003-tableS2.docx]

#Script for the GLMM analysis in Table 2

library(lme4)

library(dplyr)

library(tidyr)

BSdata<-read.table("Israel_data.csv", sep = ",", header=T)

colnames(BSdata)

#re-arrange data so that each row has data from only the first or the second brood.

#first "lengthen" to create a row for each observation of EPY number, WPY number, or Brood size--then "widen" to pull back together observations from the same male-year-brood into one row.

BSdata<-BSdata%>%

pivot_longer(cols = c("EPY_first", "BroodSize_first", "WPY_first", "EPY_second", "BroodSize_second", "WPY_second" ),

names_to = c("Variable", "BroodID"), names_pattern = "(.+)_(.+)")%>%

pivot_wider(names_from=Variable, values_from=value)

#create a new variable that is the combination of male and year

BSdata$Male_year<-paste(BSdata$Male, BSdata$year, sep = "_")

BSdata$PctWPY<-BSdata$WPY/(BSdata$BroodSize)

#define reference category:

BSdata$BroodID2<-as.factor(BSdata$BroodID)

BSdata$BroodID2<-relevel(BSdata$BroodID2, ref = "first")

BSdata$Treatment2<-as.factor(BSdata$Treatment)

BSdata$Treatment2<-relevel(BSdata$Treatment2, ref = "CC")

#to test overall effects of an interaction or multi-level categorical variable, run model with and without that variable and then compare AIC

Pat0<-glmer(cbind(WPY, EPY)~BroodID2*Treatment2+

(1|Male_year/BroodID),data=BSdata,

family = binomial, control=glmerControl(optimizer="bobyqa"))

Pat0b<-glmer(cbind(WPY, EPY)~BroodID2+Treatment2+

(1|Male_year/BroodID),data=BSdata,

family = binomial, control=glmerControl(optimizer="bobyqa"))

anova(Pat0, Pat0b)

AIC(Pat0, Pat0b)

summary(Pat0)
